# On the Course of Pernicious Anæmia in Older Persons

**Published:** 1913-06

**Authors:** J. Michell Clarke

**Affiliations:** Professor of Medicine, University of Bristol; Physician, Bristol General Hospital.


"*Scire est nescire, nisi id me*
*Scire alius sciret.*"

JUNE, 1913.

### ON THE COURSE OF PERNICIOUS ANÆMIA IN OLDER PERSONS.

BY

J. MICHELL CLARKE, M.A., M.D. Cantab., LL.D. Bristol, F.R.C.P.,

*Professor of Medicine, University of Bristol;*
*Physician, Bristol General Hospital.*

---

In a disease like pernicious anæmia, which after all is not very common, it is difficult for one observer to get a sufficient number of examples from which to draw any general conclusions from his personal observations. Moreover, in hospital work, the opportunity to follow the course of a chronic disease for any considerable time is rare, and especially so in pernicious anæmia, in which the course of the illness is characterised by marked exacerbations, during which the patient comes into hospital and is lost to sight during the intervals. To get a full knowledge of pernicious anæmia frequent observations on the blood are necessary throughout the course of the illness, including the remissions. The majority of cases of pernicious anæmia occur between the ages of 35 to 55 years, but for some time I had noticed in a few cases in elderly people that the disease appeared to follow a somewhat different course with especial features in the blood-changes, yet as they were not observed during long, continuous periods, it was possible that the variations observed might be only individual and accidental. Lately, however, I have had the opportunity of closely following the course of two cases in elderly men for about twelve months in each case, and in a third for six months.

In this paper I propose to deal with some features of pernicious anæmia where it occurs in elderly or old persons, of which I have eight cases available, and to compare them with cases occurring between the ages of 30 and 50, of which I have taken for comparison fifteen recent cases. The observations rest on a very large number of blood-counts, made or verified personally, and of which only the averages can be given in this paper. The hæmoglobin was estimated by Gowers's method or by Haldane's modification. Leishman's stain was used for films, supplemented by Jenner's stain when it seemed desirable. I do not propose to discuss at length what is and what is not true pernicious anæmia, other than to say that the evidence is in favour of its being a disease of the marrow brought about by the action of toxins varying in nature, the features of the disease being more or less constant and easily recognisable, due not to actual identity of the exciting cause in each case, but to the fact that the cause acts always primarily upon one and the same highly-specialised tissue, the marrow, which responds in a constant way. The most important changes which give the disease its characteristic clinical features are of course found in the blood, and of these the high colour index and the presence of macrocytes in large numbers seem to me the most important single changes, because the most constant, and because they are not regularly found in other forms of anæmia. To get their full significance, however, one or two blood-counts in a particular case are not sufficient, as occasionally the colour-index, especially in chronic cases, falls below 1. I am also of opinion that there is still something to be learnt as to the nature of pernicious anæmia from the variations in the number and variety of the leucocytes, and I have paid especial attention to this point.

In the type of the disease in elderly persons, which conforms to what has been described as the chronic type of pernicious anæmia, but which, though during the main part of its course it is of moderate severity, is not extremely protracted in duration, the etiology is as obscure and the onset as insidious as in other forms. In such persons most of the teeth have often been lost, and the remaining ones, especially the lower incisors, are loose with receding gums. In one case only the patient had had definite pus-formation, but this had been cured by extraction of the teeth and vaccines twelve months before the appearance of the distinctive anæmia. In three others without evidence of pus-formation cultures taken deeply from the root of the loosened teeth, after carefully cleansing the gums and superficial parts, only gave the presence of micro-organisms which Dr. Scott Williamson, pathologist to the hospital, considered were not pathogenic. Removal of the teeth had no effect on the course of the anæmia. In the other cases the teeth and gums were healthy, and this corresponds to my experience of pernicious anæmia generally—that the condition of the teeth is sometimes good, sometimes bad, but too variable to be of absolute significance in the origin of the disease. In several of the cases the patients were in easy circumstances, leading healthy lives under good hygienic conditions.

The duration in these cases is about twelve months, and the anæmia is definitiely established by the time the patient seeks medical advice. At first there is not the great loss of strength which occurs early and is so marked a symptom in the more acute forms of pernicious anæmia, and for a long time the patient is able to get about and even to do a moderate amount of work without great fatigue and in fair comfort, except for some shortness of breath and palpitation. After a few months, however, loss of strength becomes marked, and then steadily increases until the fatal termination, which occurred in six of the eight cases ; one I could not trace.

Although there are intervals in which the condition, both general and of the blood, improves, there are not the marked remissions which are so characteristic a feature of the more common type of pernicious anæmia, and on the whole, with occasional intervals of improvement, the course of the disease is gradually but steadily progressive. Gastro-intestinal symptoms, which are so frequent in all forms of pernicious anæmia, so difficult to treat, and whose frequency and severity are of such great importance to the progress of the case, are especially prominent in these cases, in the form of attacks of vomiting and diarrhœa, and especially of nausea, dryness of the mouth, and profound anorexia. This dryness of the mouth and anorexia are particularly difficult of relief. Probably, chiefly from these causes, there is a steady loss of flesh in the latter months of the illness, which becomes rapid towards the end. Beyond some tingling and numbness in feet and legs, signs of spinal affection were present in only one of these cases.

The patients show early and markedly the characteristic lemon-coloured tint of skin, and often a certain amount of scattered pigmentation. Hemorrhages do not occur except in the form of retinal hemorrhages, which were present in my cases. The spleen was either not enlarged or very slightly so. In ordinary and more acute cases of pernicious anæmia I regard the presence of a well-marked urobilin band in the spectroscopic examination of the urine as a valuable confirmatory aid in the diagnosis, but in these more chronic cases it was only occasionally found. Towards the end of the illness the blood-pressure is apt to fall very low, possibly indicating exhaustion of the supra-renals. As to the condition of the blood in these eight cases the changes are of moderate severity, corresponding to those of the more chronic type of pernicious anæmia, and is given in the third line (*c*) of the Table II, p. 105, where it is seen that the red cells average about two millions, hæmoglobin 40 per cent., total leucocytes 5720 per c.m., of which 35 per cent. are small lymphocytes and 9.5 large mononuclears. In line (*e*) two cases are given separately, as blood-counts were continuously taken at intervals of a fortnight during nine and twelve months respectively, and in (*d*) the six other cases, which were only seen for shorter periods. The condition of the blood in the prelethal stage (one week or so before death) is given separately for all these cases in line (*g*). The difference between (*c*), (*d*) and (*e*) illustrates what was said in the beginning as to the necessity of more continuous observations on the blood in cases of anæmia than we are able to secure in hospital practice, where the patients come in for short periods when they are at their worst, and also in private practice, where ordinarily the blood is only examined occasionally at arbitrary intervals. It follows from this that (*d*), taken from patients discontinuously observed, gives too unfavourable a representation of the blood-state during the whole course of the illness, and that the average of red cells, hæmoglobin and leucocytes is seen to be higher when the cases continuously observed are taken into account.

The accompanying chart, Table I, brings out this point, and shows the course of the disease in the two cases continuously followed, an average being taken for each month. The chief points are that the red corpuscles and hæmoglobin follow corresponding curves and remain at a low, though not a very low, level during the whole course of the illness, and without any great remissions or variations until near the end, when they both show a rapid and steady fall until death occurred.

The curve of the small lymphocytes is also noteworthy, as it fairly closely follows that of the red cells and hæmoglobin, and temporary improvements in the case coincided with an increase in the percentage of small lymphocytes. I have noticed this also in myeloid leukæmia, improvement under different forms of treatment coinciding with an increase of these cells. Of course, it is to be borne in mind that in nearly all cases of pernicious anæmia there is a leucopenia, together with a relative increase in the lymphocytes and mononuclears, especially in the more chronic cases, and allowance must be made for this.

There are extraordinary variations in the total leucocytes for which I could not account, and which could not be accounted for by any variation in the clinical symptoms, nor do they correspond with the small variations in amounts of red cells and hæmoglobin. There is a marked rise of leucocytes in each case shortly before death. The polymorphonuclears show a remote correspondence with the variations in total number of leucocytes.

### TABLE I.

| Case A. | Red Cells. | Hæmo-globin. | Colour Index. | Total Leucocytes. | Polymorpho-nuclears. | Small lymphocytes. | Large lymphocytes and mononuclears. | Eosinophils. |
|---|---|---|---|---|---|---|---|---|
| | Per c.m. | % | % | Per c.m. | % | % | % | % |
| 1911. Sept. .. .. | 1,700,000 | 50 | 1.4 | 8,000 | 53 | 34 | 18 | 4 |
| Oct. .. .. | 2,100,000 | 50 | 1.2 | 7,600 | 72 | 16 | 10 | 2 |
| Nov. .. .. | 3,700,000 | 60 | .8 | 7,500 | 62 | 22 | 14 | 1 |
| Dec. .. .. | 3,100,000 | 60 | 1 | 10,000 | 59 | 29 | 7.4 | .6 |
| 1912. Jan. .. .. | 2,500,000 | 54 | 1.+ | 10,000 | 69 | 23 | 7 | .3 |
| Feb. .. .. | 3,800,000 | 70 | 1.+ | 12,000 | 62 | 25 | 12 | .2 |
| March 5th | 2,400,000 | 45 | .9 | 4,500 | 49 | 39 | 11 | .2 |
| March 20th | 1,100,000 | 28 | 1.3 | 10,700 | 69 | 27 | 3 | 0 |
| Case B. | | | | | | | | |
| 1911. Nov. .. .. | 2,800,000 | 62 | 1.1 | 8,400 | 42.5 | 42 | 12 | 1.5 |
| Dec. .. .. | 3,000,000 | 70 | 1.1 | 7,000 | 39.5 | 45.5 | 15 | 1 |
| 1912. Jan. .. .. | 3,200,000 | 70 | 1 | 6,000 | 42 | 49 | 7.5 | 1 |
| Feb. .. .. | 3,700,000 | 70 | .9 | 6,000 | 51 | 35.5 | 10.5 | 1.5 |
| March .. .. | 3,100,000 | 68 | 1.1 | 11,500 | 39 | 38 | 19 | 3 |
| April .. .. | 2,200,000 | 60 | 1.3 | 5,000 | 36 | 44.5 | 17 | 1.5 |
| May .. .. | 2,900,000 | 60 | 1 | 4,300 | 37 | 43 | 16 | 2 |
| June .. .. | 2,800,000 | 62 | 1.1 | 9,000 | 64 | 20 | 14.5 | 2 |
| July .. .. | 3,400,000 | 55 | .8 | 9,200 | — | — | — | — |
| August .. | 1,300,000 | 28 | 1.1 | 6,500 | 65 | 18 | 12 | 2 |
| Sept. .. .. | 860,000 | 25 | 1.4 | 3,400 | 54 | 38 | 7.5 | 0 |
| Oct. 5th .. | 912,000 | 25 | 1.4 | 10,000 | 52 | 33 | 12 | 0 |

Possibly these variations indicate an attempt on the part of the organism to react to the entrance of fresh doses of toxin.

Both these patients were treated at intervals with 10 to 20 c.c. doses of polyvalent antistreptococcic serum per rectum, but I could not trace out any effect from these injections on either the number or the percentage variety of the leucocytes.

To return to the eight cases taken together. The large mononuclears, though slightly increased in numbers, showed no striking variations. In these cases, however, these cells were generally of great size, and had large and very irregular nuclei, the nuclei having many lobes and often several vacuoles in them, the whole giving the impression that the cells were in a very active state. These are amœboid cells, and possibly show an attempted reaction on the part of the organism to infections or poisons with which the polymorphonuclears are unable to deal.

An important point about these cases in elderly persons is that both megaloblasts and normoblasts are either absent throughout the course of the disease until just before the end or present in very scanty numbers. This is true of chronic cases of pernicious anæmia generally, according to Gulland and Goodall, and is certainly a marked feature of the cases under consideration. This is one of the chief differences from pernicious anæmia in younger patients, or in the more ordinary acute or subacute forms, and might lead to an error of diagnosis if the presence of megaloblasts be taken as the criterion of the disease. Again, from the point of view of those who look upon pernicious anæmia as essentially a megaloblastic anæmia, this point might be considered sufficient to exclude these cases from the category. However, the fact that in the late stages there is an irruption of these cells into the blood, often in large numbers, thus bringing the condition of the peripheral blood into conformity with the general type, is a sufficient answer to this objection, for there could be no difference of opinion as to the films taken in the final stages presenting the blood-picture of megaloblastic pernicious anæmia. Macrocytes are numerous throughout, and my experience confirms the statement of Buckmaster, that of the abnormal changes in the blood this is the most constant in all forms of pernicious anæmia; I would add in association with a high colour-index.

Blood platelets are scanty in these cases. Chromatoplasia and granular degeneration of the red corpuscles, though generally present at intervals and towards the end, are not marked features. A few myelocytes, 1 per cent. or less, are found. In Table II (*c*) is given the condition of the blood in these eight cases, and in (*a*) that of fifteen cases of pernicious anæmia below the age of fifty, several of them of acute type, based on an average of numerous blood-counts taken in all phases of the disease. It is seen that they differ in the following points.

In the former (*c*) the number of red cells is greater, percentage of hæmoglobin slightly higher, not so much in proportion as the number of red-cells, and the colour-index lower, whilst the number of leucocytes is much greater, and the relative proportion of small lymphocytes and mononuclears is also higher. The contrast as regards leucocytes and lymphocytes would be even more marked but that the leucopenia happens in the fifteen cases in (*a*) to be less than the average usually given for ordinary cases of pernicious anæmia.

The contrast between the two types becomes far more striking, except as to one point, if the blood-state of the eight cases in (*c*) is compared with that in (*b*), which represents the average of a number of blood-counts from cases during the severe exacerbations which characterise the course of ordinary acute and subacute pernicious anæmia, and at the same time we remember that such severe exacerbations were absent in the eight older and more chronic cases (*c*). In the exacerbations the red cells and hæmoglobin, as compared with (*c*), are now reduced still further, to about half, the colour-index is correspondingly higher, and the leucopenia very marked. On the other hand, there is one point in whiich the two conditions now approximate, and that is in the proportion of lymphocytes present, for these show a temporary but marked increase during exacerbations of the disease. In both (*a*) and (*b*), especially in the latter, the difference between them and (*c*) in the matter of megaloblasts and normoblasts is noteworthy.

### TABLE II.

| | Red cells. | Hæmoglobin. | Index. | Total Leucocytes. | Polymorphonuclears. | Small lymphocytes. | Large lymphocytes and mononuclears. | Eosinophils. | Basophils. | Macrocytes. | Megaloblasts. | Normoblasts. | |
|---|---|---|---|---|---|---|---|---|---|---|---|---|---|
| | | % | % | | % | % | % | % | % | | | | |
| (*a*) Blood from average of 15 cases under 50 years of age. | 1,575,000 | 36.8 | 1.2 —1.3 | 4,500 | 67 | 24 | 6.7 | .9 | .4 | numerous. | | | (*a*) |
| (*b*) The same during severe exacerbations. | 964,000 | 24 | 1.3 | 2,400 | 52.9 | 42 | 4 | .3 | .2 | numerous | about 20% | numerous. | (*b*) |
| (*c*) Blood from average of 8 cases over 55 years of age. | 2,120,000 | 40 | 1 | 5,720 | 53 | 35 | 9.5 | 2.4 | .4 | ,, | few or absent. | | (*c*) |
| (*d*) Blood from average of 6 cases in (*c*) over 55 years of age. | 1,754,000 | 35 | 1+ | 5,000 | 55 | 34 | 8 | 2.7 | .5 | ,, | absent or very few. | | (*d*) |
| (*e*) The same in 2 cases followed for last 12 months of life. | 2,680,000 | 48 | .9 | 7,800 | 60 | 29 | 9.4 | 1.4 | .2 | ,, | .3% | .2% | (*e*) |
| | 2,800,000 | 59 | 1.1 | 8,300 | 46 | 37.5 | 13.5 | 1.8 | .4 | ,, | absent. | .4% | |
| (*f*) The blood in prelethal state (within 48 hours of death) in cases under 50 years of age. | 850,000 | 17 | 1.8 | 2,400 | 54 | 35 | 10.2 | .2 | .2 | ,, | 10-40% | numerous. | (*f*) |
| (*g*) The same in prelethal states in cases over 55 years of age. | 1,000,000 | 25 | 1.3 | 9,700 | 55 | 31 | 12 | .3 | .4 | ,, | 12% | 3.5% | (*g*) |

The blood-state in severe exacerbations may be as bad as or worse than that seen in the prelethal stage of pernicious anæmia, *i.e.* within twenty-four to forty-eight hours of death, and it is interesting to note that patients may recover from such exacerbations and afterwards enjoy a long remission of comparatively good health, and that they often die with a condition of blood not so bad as it may have been in a previous exacerbation from which they made a good recovery.

I have had the opportunity of examining the blood in a number of cases of both the acute and chronic (or senile) type within twenty-four to forty-eight hours of death. The condition of the blood may then be taken to represent the ultimate result of the disease on the marrow. However great the differences in the blood between the two forms during the previous course of the disease in the prelethal stage they closely approximate. The table (*f*) (*g*) gives the results in the two types. There is still a difference in severity, but now slighter as regards red cells, hæmoglobin and colour-index ; the chief difference is in the total leucocyte count, which rises towards death in the chronic or senile cases ; the relative numbers of the varieties of leucocytes closely approximate, the percentage of lymphocytes and mononuclears being high in both; and there is now an irruption of megaloblasts and normoblasts into the blood in the chronic cases. This took place during the last two or three weeks of life in my cases and increased until death. Thus, as stated above, in the final stage of all forms of pernicious anæmia the blood-state is practically identical, and it follows that blood examinations made then would not show the differences found earlier.

Thus in the final stage of the type of case under consideration the blood-changes at last present the full picture of that of pernicious anæmia, with the difference that runs through the course of the case that there is no marked leucopenia and even a slight increase in the number of leucocytes. That this difference and the greater variations in the leucocyte count than occur in acute or subacute cases in younger patients may be due either to a different etiology or to a difference in reaction on the part of the marrow is obvious. There is no evidence to decide between these alternatives. Whatever the poison at work, it would seem to be combated rather by the lymphocytes and large mononuclears than by the polymorphonuclears.

A great deal can be done by treatment in mitigating the course and severity of the disease, though unfortunately the effects are not lasting. When the patient first comes under observation rest in bed is the first essential point, and next careful regulation of the diet with appropriate remedies for gastro-intestinal disturbances if these are present, as is generally the case. It must be seen that a sufficient amount of easily-digested food is taken, and the diet should include green vegetables and the juices of fruit. It need hardly be said that arsenic is the drug from which most benefit is derived, given not continuously but for definite periods, in gradually-increasing doses. In the type of case under consideration iron, preferably in the form of one of the so-called organic preparations, is certainly useful.

Several of the patients were treated from time to time with serum, and showed a marked improvement under it, in the earlier stages of the illness. This was manifested by an improved general sense of well-being and gain in strength, corresponding with amelioration in the state of the blood. As stated above, however, I could not connect any definite feature of the blood with the use of serum. It was not given in relation to the presence or absence of pyorrhœa. At first I used antistreptococcic polyvalent serum, later horse serum. It was given per rectum in doses of 10 to 15 c.c. daily for one to two weeks, then every other day for two to four weeks.

Two cases were treated by salvarsan ; the one, a man, aged 62, showed a remarkable improvement. A mean of several counts, the blood before salvarsan, gave red cells 1,150,000, hæmoglobin 32 per cent., index 1.4, leucocytes 6,300, megaloblasts and monoblasts 2.4 per cent. Salvarsan was injected on July 6th. After steady improvement and gain of weight the counts gave on July 30th reds 4,200,000, hæmoglobin 65 per cent., index .8, leucocytes 10,700, no nucleated red cells.

Unfortunately I have not been able to trace this patient, so that I cannot say whether he has remained well. In the other case there was little benefit.

---

### METHODS OF DIAGNOSIS IN GASTRIC CANCER.

BY

J. M. Fortescue-Brickdale, M.A., M.D. Oxon.,

